# Association between maternal smoking during pregnancy and attention-deficit/hyperactivity disorder (ADHD) in children aged 4–15 years: A secondary data analysis from the NHANES dataset

**DOI:** 10.18332/tid/207094

**Published:** 2025-08-08

**Authors:** Baomei He, Junxiong Peng, Mengqi Wu, Yanbing Deng, Ying Zhang

**Affiliations:** 1 Center for Reproductive Medicine, Department of Pediatrics, Zhejiang Provincial People’s Hospital (Affiliated People’s Hospital), Hangzhou Medical College, Hangzhou, China; 2 Department of Pediatrics, Xianju People’s Hospital, Zhejiang Southeast Campus of Zhejiang Provincial People’s Hospital, Affiliated Xianju’s Hospital, Hangzhou Medical College, Xianju, China

**Keywords:** maternal smoking during pregnancy, attention-deficit/hyperactivity disorder (ADHD), children, sex differences, National Health and Nutrition Examination Survey

## Abstract

**INTRODUCTION:**

The association between maternal smoking during pregnancy (MSDP) and the risk of attention-deficit hyperactivity disorder (ADHD) in children remains inconclusive. This study aims to investigate the relationship between MSDP and ADHD in children aged 4–15 years, with a particular focus on sex differences.

**METHODS:**

This secondary analysis used cross-sectional data from the 1999–2002 National Health and Nutrition Examination Survey (NHANES), comprising 5548 US children and adolescents aged 4–15 years. Inclusion criteria comprised complete maternal smoking history during pregnancy and parent-reported ADHD diagnosis data. Multivariable logistic regression models were employed to assess the association between MSDP and ADHD, with further stratified analyses.

**RESULTS:**

Multivariable logistic regression analysis identified a significant association between MSDP exposure and elevated odds of ADHD (OR=2.11; 95% CI: 1.48–3.00). Sex-stratified analyses revealed that this association was more pronounced in female children (OR=4.18; 95% CI: 2.17–8.03) than in male children (OR=1.64; 95% CI: 1.13–2.38), the interaction between groups was significant (p for interaction <0.01).

**CONCLUSIONS:**

MSDP showed a statistically significant association with higher ADHD odds, with more pronounced estimates observed in females compared to males.

## INTRODUCTION

Attention-deficit/hyperactivity disorder (ADHD) is a pervasive and debilitating disorder characterized by persistent attention deficit, hyperactive-impulsivity, or both, which imposes a significant burden on individuals and society^1^. A substantial body of research has indicated that ADHD manifests in 3–10% of the population^2^. The etiology of ADHD is thought to be multifactorial, with environmental and genetic influences or complex interactions being posited as potential contributors^3-5^. However, further study is required to clarify the factors involved and their individual contributions^3,4^. The life-course and DOHaD (Developmental Origins of Health and Disease) concepts suggest that maternal environmental exposures can negatively impact offspring health, with effects lasting into adulthood^6,7^.

Smoking during pregnancy affects about 1.7% of pregnancies globally. Ireland, Uruguay, Bulgaria, and the United States have the highest prevalence, with smoking rates of 38.4%, 29.7%, 29.4%, and 25%, respectively^8^. But only a small percentage of these individuals quit after learning of their pregnancy^9^. Evidence has elucidated potential mechanisms linking maternal smoking during pregnancy (MSDP) to neurodevelopmental impairments: 1) transplacental nicotine, carbon monoxide, and polycyclic aromatic hydrocarbons induces cellular toxicity; 2) hypoxia from vasoconstriction-induced placental ischemia and nicotinic acetylcholine receptor (nAChR) signaling disruption, impairing cholinergic neurotransmission; and 3) epigenetic dysregulation (altered DNA methylation at neurodevelopmental loci and miRNA-mediated modulation) disrupting gene expression^10-12^.

MSDP has garnered increasing attention as a modifiable environmental risk factor in this context. Research has demonstrated that harmful substances present in maternal smoking can exert a detrimental effect on neurodevelopment^13-15^. Chronic nicotine exposure during pregnancy has also been linked to reduced attention span and hyperactivity in offspring, according to animal studies^16,17^. However, other investigators have found no increased risk of ADHD in children whose mothers smoked during pregnancy^18,19^.

ADHD prevalence is higher in males, yet under-diagnosed in females globally^2,14^. Differences in the relationship between MSDP and ADHD by gender remains to be elucidated^14,20,21^. Research indicates that MSDP primarily increases hyperactivity and aggression in males^22^, but methodological and sample limitations persist^21^. Two Saguenay Youth Study investigations found that MSDP was associated with decreased regional brain volume, especially in female offspring. Researchers identified reduced thickness in the frontal, temporal, and parietal regions (including the middle frontal, lateral orbitofrontal, and parahippocampal cortices, which are known for their high density of nicotinic receptors). These effects were most pronounced in exposed females across all regions, with the exception of the parietal areas and the parahippocampal cortices^23,24^.

Utilizing data from the National Health and Nutrition Examination Survey (NHANES) with enhanced covariate adjustment (including socioeconomic indicators, environmental and perinatal factors), this study examined the association between MSDP and ADHD in US children, with particular focus on sex-specific differences.

## METHODS

### Study design

This study employed data from the NHANES, a continuous national survey administered by the National Center for Health Statistics (NCHS) of the Centers for Disease Control and Prevention (CDC). The purpose of NHANES is to collect comprehensive health and nutrition information from a representative sample of the US civilian non-institutionalized population. Data collection began in the early 1960s and has continued since 1999, with approximately 5000 participants sampled annually from diverse geographical regions. Ethical approval for this investigation was granted by the NCHS Ethics Review Committee, and informed consent was obtained from all participants, with parental or guardian consent required for those under 18 years of age.

### Study sample

**Figure 1:**
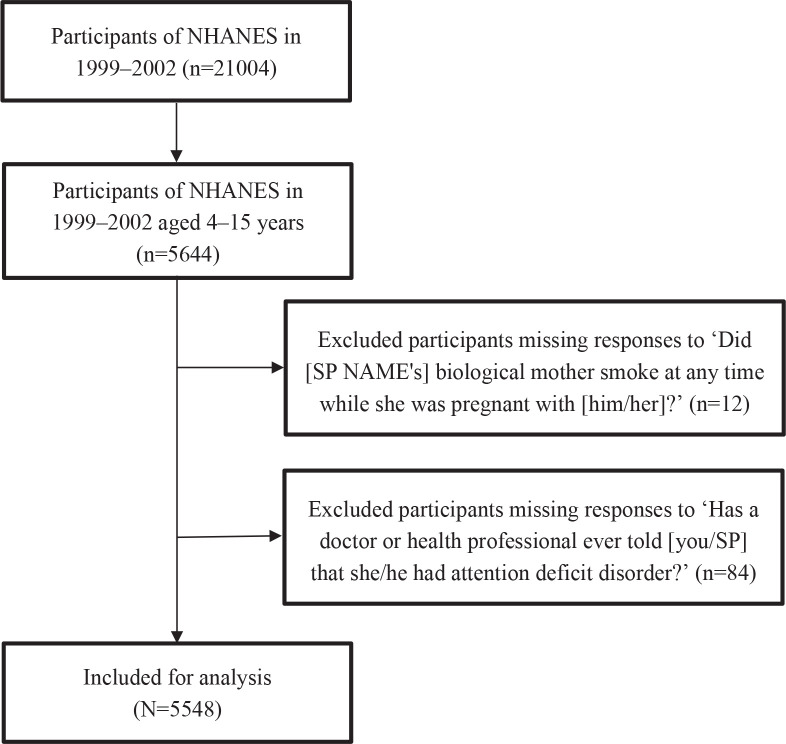
Flow chart of participants

We used NHANES data from 1999 to 2002, which included comprehensive ADHD assessments in children. A lower age limit of 4 years was implemented due to the fact that inquiries concerning ADHD were exclusively posed to children aged ≥4 years. Conversely, an upper age limit of 15 years was established because the inquiry regarding maternal smoking was posed only to children up to the age of 15 years. From an initial 5644 eligible participants, we excluded 12 individuals with missing ADHD data and 84 with incomplete maternal smoking records, yielding a final analytical sample of 5548 participants. Figure 1 presents a flow chart that illustrates the process of participant selection, illustrating the inclusion and exclusion criteria.

### Assessment of MSDP and ADHD outcomes in offspring

Data reported by parents or guardians during NHANES interviews were used to assess MSDP and ADHD status^25^. MSDP exposure was ascertained through affirmative responses to the questionnaire item: ‘Did the biological mother smoke at any time while she was pregnant with him/her?.’ ADHD diagnosis was determined based on positive responses to: ‘Has a doctor or health professional ever told you/your spouse that she/he had attention deficit disorder?’^26^.

### Other covariates

The following covariates were analyzed: age, sex, race/ethnicity, birth weight, blood lead levels, health insurance, parental education level, Neonatal Intensive Care Unit (NICU) admission, and daycare or preschool attendance. These were categorized based on data acquisition processes in NHANES database^25^. Birth weight data were converted to grams for analysis consistency, and the birth weight variable was classified as <2500 g, 2500–4000 g, and >4000 g. Parental education level was categorized as lower than high school, high school, and higher than high school.

Blood samples were collected during physical examinations, preserved at -20°C, and transferred to a central laboratory. The protocols delineated on the NHANES website were scrupulously followed, and atomic absorption spectrophotometry utilizing quadrupole ICP-MS technology and a PerkinElmer Model SIMAA 6000 was employed to ascertain lead levels (detection limit: 0.01 µmol/L).

### Statistical analysis

Statistical analyses were performed using R^27^ and EmpowerStats software^28^. The application of sample weights followed the analytical guidelines established by the NCHS to ensure data representativeness of the non-institutionalized US population. The independent variable was MSDP, and the dependent variable was ADHD. Continuous variables are presented as mean (95% CI) and categorical variables as percentage (95%), with p-values from survey-weighted linear regression and chi-square tests, respectively. Missing covariate values exceeding 5% were processed using a dummy variable. Variables based on previous studies were incorporated as potential confounders^26,29^. Three multivariable logistic regression models were developed with results reported as odds ratios (ORs) with 95% confidence intervals: Model 1 included no adjusted covariates; Model 2 was adjusted for age, sex, and race/ethnicity; and Model 3 included all covariates displayed in Table 1. Further subgroup analyses and interaction tests were conducted to identify whether the association between MSDP and ADHD varied by age, sex, race/ethnicity, birth weight, parental education level, NICU admission status, daycare or preschool attendance, health insurance coverage, and blood lead level. A p<0.05 (two-sided) was considered statistically significant.

**Table 1:** Baseline characteristics of the study participants by ADHD outcomes in US children aged 4–15 years. NHANES 1999–2002 (weighted)

*Characteristics*	*ADHD*		p*
	*No*	*Yes*	
	*% (95% CI)*	*% (95% CI)*	
**Sex**			<0.01
Male	48.50 (46.32–50.68)	76.16 (71.10–80.57)	
Female	51.50 (49.32–53.68)	23.84 (19.43–28.90)	
**Race/ethnicity**			<0.01
Mexican American	12.78 (9.89–16.37)	4.61 (3.39–6.25)	
Non-Hispanic White	58.91 (54.20–63.47)	69.56 (62.26–76.00)	
Non-Hispanic Black	14.81 (11.47–18.93)	14.38 (11.14–18.37)	
Other	13.49 (9.94–18.06)	11.44 (6.30–19.87)	
**Birth weight** (g)			0.40
<2500	7.27 (6.29–8.40)	9.69 (6.27–14.68)	
2500–4000	82.41 (80.02–84.56)	80.41 (75.13–84.79)	
>4000	10.32 (8.66–12.25)	9.91 (6.30–15.25)	
**Parental education level**			0.91
High school or lower	24.41 (21.87–27.15)	23.61 (17.07–31.69)	
High school	27.30 (24.31–30.51)	26.41 (19.31–34.98)	
High school or higher	48.29 (44.23–52.38)	49.98 (41.54–58.42)	
**NICU admission**			0.03
No	88.37 (86.70–89.86)	83.06 (76.50–88.07)	
Yes	11.63 (10.14–13.30)	16.94 (11.93–23.50)	
**Daycare/preschool attendance**			<0.01
No	27.73 (24.50–31.21)	17.03 (10.59–26.24)	
Yes	72.27 (68.79–75.50)	82.97 (73.76–89.41)	
**Maternal smoking during pregnancy**			<0.01
No	83.08 (80.43–85.43)	68.12 (59.40–75.74)	
Yes	16.92 (14.57–19.57)	31.88 (24.26–40.60)	
**Health insurance**			0.06
No	13.90 (11.52–16.68)	9.43 (6.16–14.16)	
Yes	86.10 (83.32–88.48)	90.57 (85.84–93.84)	
	***Mean (95% CI)***	***Mean (95% CI)***	
**Age** (years)	9.40 (9.30–9.50)	10.90 (10.35–11.45)	<0.01
**Blood lead level** (μmol/L)	0.08 (0.07–0.08)	0.08 (0.07–0.09)	0.89

* For categorical variables, p-values were calculated using survey-weighted chi-squared tests. For continuous variables, p-values were calculated using survey-weighted linear regression analyses.

## RESULTS

### Baseline characteristics

Table 1 illustrates the baseline characteristics of study participants categorized by ADHD outcomes. The mean age for children in the ADHD group was 10.90 years, which was higher than the 9.40 years observed in the non-ADHD group. With respect to sex distribution, the ADHD group exhibited a significantly higher proportion of males (76.16%) compared to the non-ADHD group (48.50%). In terms of race/ethnicity, the ADHD group had a significantly higher proportion of non-Hispanic White children (69.56%), while Mexican American children had the lowest ADHD rate at 4.61%. The impact of MSDP was notable, with 31.88% of children in the ADHD group having mothers who smoked during pregnancy, compared to only 16.92% in the non-ADHD group. Furthermore, a significant disparity was observed in the NICU admission rate, with the ADHD group exhibiting a rate of 16.94%, which was substantially higher than the rate of 11.63% observed in the non-ADHD group. A similar pattern was observed in the attendance of daycare/preschool, with 17.03% of children in the ADHD group attending compared to 27.73% in the non-ADHD group. However, no statistically significant differences were observed in birth weight, parental education level, health insurance, or blood lead level.

### Association between MSDP and ADHD

Table 2 shows the association between MSDP and ADHD in offspring using three models with different levels of covariate adjustment. In Model 1, without adjustment for covariates, MSDP was associated with a significantly elevated odd of ADHD (OR=2.30; 95% CI: 1.64–3.21). When adjusted for age, sex, and race/ethnicity in Model 2, the association remained significant but slightly attenuated (OR=2.08; 95% CI: 1.50–2.89). In Model 3, which further adjusted for birth weight, parental education level, NICU admission, daycare/preschool attendance, health insurance, and blood lead level, the association persisted (OR=2.11; 95% CI: 1.48–3.00). These findings indicate that MSDP is a significantly associated with increased odds of ADHD, with a notable effect even after adjusting for multiple confounding variables.

**Table 2:** Multivariate logistic regression analysis of the association between MSDP and ADHD in US children aged 4–15 years, NHANES 1999–2002 (weighted)

	*Model 1* *OR (95% CI)*	*Model 2* *AOR (95% CI)*	*Model 3* *AOR (95% CI)*
**Maternal smoking during pregnancy**			
No ®	1	1	1
Yes	**2.30 (1.64–3.21)**	**2.08 (1.50–2.89)**	**2.11 (1.48–3.00)**

AOR: adjusted odds ratio. Model 1: no covariates were adjusted. Model 2: age, sex, race/ethnicity were adjusted. Model 3: adjusted as for Model 2 plus birth weight, parental education level, NICU admission, daycare/preschool attendance, health insurance, blood lead level. ® Reference category.

### Subgroup analysis

**Figure 2:**
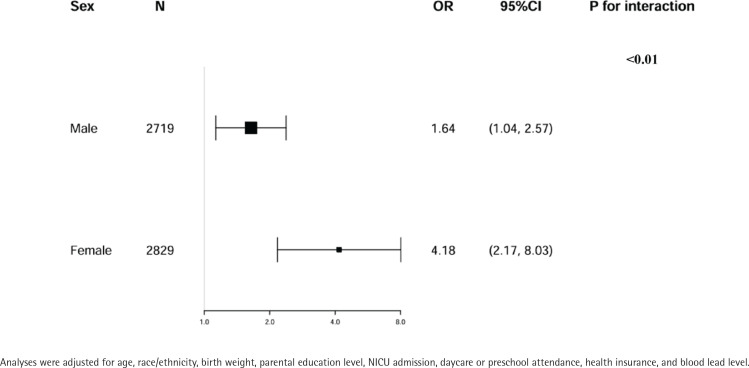
Multivariate logistic regression analysis of the association between MSDP and ADHD stratified by sex in US children aged 4–15 years, NHANES 1999–2002 (weighted)

**Figure 3:**
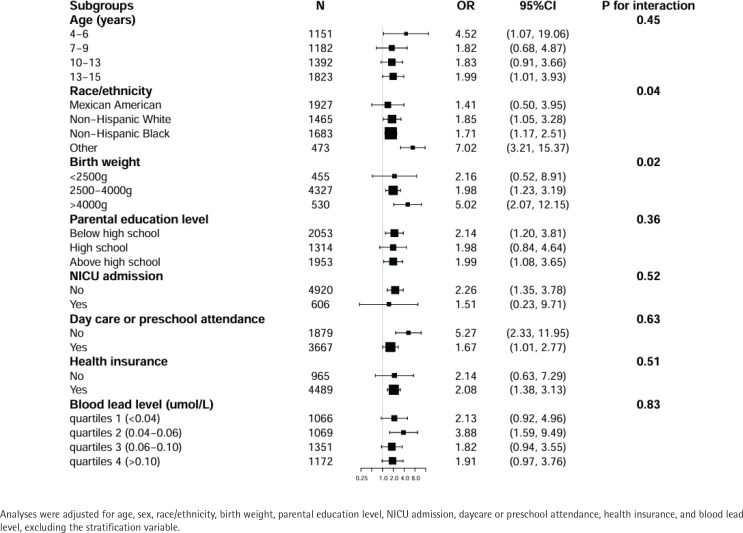
Multivariate logistic regression analysis of the association between MSDP and ADHD according to subgroups in US children aged 4–15 years, NHANES 1999–2002 (weighted)

Stratification analyses and interaction analysis were conducted, shown in Figures 2 and 3. These analyses were stratified by age, sex, race/ethnicity, birth weight, parental education level, NICU admission, daycare/preschool attendance, health insurance, and blood lead level, excluding the stratification variable. Age was categorized into four ranges: 4–6, 7–9, 10–12, and 13–15 years.

Sex subgroup analyses demonstrated a stronger association in females (OR=4.18; 95% CI: 2.17–8.03) compared to males (OR=1.64, 95% CI: 1.04–2.57), with a statistically significant interaction (p for interaction <0.01) (Figure 2).

Figure 3 shows consistent but quantitatively varying effect sizes across subgroups, with statistically significant associations between MSDP and ADHD observed in: 1) children aged 4–6 years (OR=4.52; 95% CI: 1.07–19.06) and 13–15 years (OR=1.99; 95% CI: 1.01–3.93); 2) Non-Hispanic White (OR=1.85, 95% CI: 1.05–3.28), Non-Hispanic Black (OR=1.71; 95% CI: 1.17–2.51), and Other racial/ethnic groups (OR=7.02; 95% CI: 3.21–15.37); 3) participants with birth weight 2500–4000 g (OR=1.98; 95% CI: 1.23–3.19) and >4000 g (OR=5.02; 95% CI: 2.07–12.15); 4), those with parental education both below (OR=2.14; 95% CI: 1.20–3.81) and above (OR=1.99; 95% CI: 1.08–3.65) high school level; 5) children without NICU admission (OR=2.26; 95% CI: 1.35–3.78); 6) participants with health insurance coverage (OR=2.08; 95% CI: 1.38–3.13); and 7) those with blood lead level 0.04–0.06 µmol/L (OR=3.88; 95% CI: 1.59–9.49). The association patterns were similar regardless of daycare/preschool attendance status.

## DISCUSSION

This study, based on NHANES data, examined the association between maternal smoking during pregnancy (MSDP) and attention-deficit/hyperactivity disorder (ADHD) in children, with a particular focus on sex differences. The results highlight the significant association between MSDP and ADHD, providing evidence to support the hypothesis that MSDP is an important potential risk factor for ADHD. Notably, this association was more pronounced in female children than in male children.

Our results indicate that MSDP significantly increases the likelihood of ADHD in children, even after adjustment for multiple confounders. This is consistent with previous studies suggesting that *in utero* exposure to nicotine and other harmful chemicals in tobacco contributes to adverse effects on the fetal central nervous system^30,31^. Nicotine may impair fetal dopaminergic neural pathways, a biological mechanism directly implicated in the pathophysiology of ADHD^9^.

ADHD is generally more common in males; females are often underdiagnosed and may have less obvious but equally disabling symptoms such as inattention^32^. A striking finding of this research is the sex difference in the effect of MSDP on ADHD. These results should be considered in light of the potential association between MSDP and ADHD, which may be stronger in females. However, this finding requires further investigation to be confirmed. Fetal hormonal differences in males and females may lead to differential susceptibility to the harmful effects of nicotine and other chemicals in tobacco^33,34^. Sex-linked genetic factors may also play a role in how exposure to toxic substances affects development^35^. In addition, genetic susceptibility with sex-specific expression patterns may also play a role, highlighting the importance of investigating the biological mechanisms underlying this disparity in future studies.

Our analysis showed that the effect of MSDP on ADHD varies between racial/ethnic groups. The environmental and socioeconomic contexts that influence maternal smoking behaviors may differ by race/ethnicity. This is consistent with previous research on cultural and contextual determinants of maternal health behaviors and child development^36^.

Infants with a birth weight >4000 g exhibited the strongest association across different birth weight groups, as evidenced by an odds ratio (OR) of 5.02. This suggests that macrosomia may amplify the adverse effects of MSDP on neurodevelopmental outcomes, potentially due to synergistic interactions between maternal smoking and metabolic conditions associated with high birth weight, such as maternal diabetes or gestational obesity. Interestingly, infants with a birth weight <2500 g did not demonstrate a statistically significant association with ADHD. The lack of significance in low-birth-weight infants (<2500 g) may be attributed to the smaller sample size in this subgroup, which could have limited the statistical power to detect significant associations. Alternatively, it may suggest that other factors, such as neonatal intensive care or early interventions, mitigate the impact of MSDP in this population.

These findings underscore the clinical relevance of addressing MSDP as a potentially modifiable exposure associated with ADHD. The observed sex-specific differences – with stronger associations in female offspring – call for heightened clinical vigilance in monitoring potential ADHD symptoms among prenatally exposed girls. Furthermore, the nationally representative NHANES sample enhances generalizability to the US pediatric population, while the inclusion of covariates – including blood lead levels, daycare attendance, socioeconomic indicators, and perinatal factors – addresses critical environmental and clinical confounders.

### Limitations

Several important limitations warrant cautious interpretation of the findings. Firstly, the cross-sectional NHANES design precludes causal inference, and residual confounding persists despite adjustment for key covariates. Unmeasured genetic predispositions and environmental exposures may contribute to observed associations. Secondly, MSDP data were self-reported, potentially introducing: 1) recall bias due to retrospective reporting, 2) misclassification bias from lack of smoking frequency and trimester-specific data, and 3) social desirability bias leading to underreporting. Thirdly, the binary classification of ADHD based on caregiver-reported diagnoses may be biased by over-reporting tendencies. The combined measurement limitations of MSDP and ADHD variables warrant cautious interpretation of the observed ORs.

## CONCLUSIONS

This cross-sectional study demonstrates a significant association between maternal smoking during pregnancy (MSDP) and ADHD in school-aged children, with notably stronger associations observed among female offspring. These findings suggest that MSDP reduction could be considered as part of comprehensive ADHD prevention strategies, though confirmation through longitudinal studies with biochemical exposure validation is required. Future studies should employ prospectively collected smoking data with biomarker verification, and elucidate the potential neurodevelopmental mechanisms underlying these sex-specific associations.

## Data Availability

The data supporting this research are available from the following source: http://cdc.gov/nchs/nhanes
